# Using artificial intelligence algorithms to reconstruct the heat transfer coefficient during heat conduction modeling

**DOI:** 10.1038/s41598-023-42536-w

**Published:** 2023-09-15

**Authors:** Elzbieta Gawronska, Maria Zych, Robert Dyja, Grzegorz Domek

**Affiliations:** 1 Faculty of Mechanical Engineering and Computer Science, Czestochowa University of Technology, Dabrowskiego 69, 42-201 Czestochowa, Poland; 2 Faculty of Mechatronics, Kazimierz Wielki University, Jana Karola Chodkiewicza 30, 85-064 Bydgoszcz, Poland

**Keywords:** Mechanical engineering, Computational science, Computer science

## Abstract

The article shows the usage of swarming algorithms for reconstructing the heat transfer coefficient regarding the continuity boundary condition. Numerical calculations were performed using the authors’ own application software with classical forms of swarm algorithms implemented. A functional determining error of the approximate solution was used during the numerical calculations. It was minimized using the artificial bee colony algorithm (ABC) and ant colony optimization algorithm (ACO). The considered in paper geometry comprised a square (the cast) in a square (the casting mold) separated by a heat-conducting layer with the coefficient $\kappa$. Due to the symmetry of that geometry, for calculations, only a quarter of the cast-mold system was considered. A Robin’s boundary condition was assumed outside the casting mold. Both regions’ inside boundaries were insulated, but between the regions, a continuity boundary condition with nonideal contact was assumed. The coefficient of the thermally conductive layer was restored using the swarm algorithms in the interval $900{-}1500 \; [\mathrm{W/m}^{2}\textrm{K}$] and compared with a reference value. Calculations were carried out using two finite element meshes, one with 111 nodes and the other with 576 nodes. Simulations were conducted using 15, 17, and 20 individuals in a population with 2 and 6 iterations, respectively. In addition, each scenario also considered disturbances at 0$\%$, 1$\%$, 2$\%$, and 5$\%$ of the reference values. The tables and figures present the reconstructed value of the $\kappa$ coefficient for ABC and ACO algorithms, respectively. The results show high satisfaction and close agreement with the predicted values of the $\kappa$ coefficient. The numerical experiment results indicate significant potential for using artificial intelligence algorithms in the context of optimization production processes, analyze data, and make data-driven decisions.

## Introduction

AI has been instrumental in addressing various complex issues, with road safety being a prime example. Adaptive safety systems have been developed to enhance the security of moving vehicles, often requiring rapid prediction of potential dangers to avert accidents or traffic incidents. Meier et al.^1^ introduced an AI-based method that automatically learns a predictive function. These models demonstrate improved performance and increased passenger security when integrated with adaptive safety systems.

Swarm algorithms, inspired by biological observations of animals such as bee swarms, ant colonies, bird flocks, and worm groups, have made significant advancements in AI. Hackwood^2^ presented the concept of intelligent swarming, emphasizing the adaptability of these algorithms to various constraints, including space size and independence of the number of variables.

As we transition into Industry 4.0, the appropriate application of AI algorithms becomes increasingly crucial.

One such application involving the heat conduction problem with an unknown heat source was discussed by Karaboga^3^. The author formulated this physical problem as an optimization challenge, using heuristic algorithms, such as genetic algorithms, to search for improved solutions. These algorithms utilize evolutionary mechanisms and natural selection principles to navigate available solutions.

The swarming algorithms were modeled on the intelligence of bees. Karaboga^4^ proposed the Artificial Bee Colony (ABC) algorithm, a model based on the food-seeking behavior of honey bees. The model encompasses three main elements: employed bees, food sources, and non-employed bees. Additionally, bees use an extended dance technique to enhance communication about food sources with other individuals.

In the conventional approach, optimization problems are typically addressed through gradient-based methods. Gradient-based algorithms and bee/ant swarm algorithms represent distinct methodologies for optimization. The first of them leverage the derivatives of a function to find the value where the function attains its smallest value. Whereas the second one, to ascertain the most optimal solution, employs natural evolution as a source of inspiration, drawing from the evolutionary processes observed in living species within the natural environment. No gradient counting is needed in ABC and ACO algorithms.

Gradient-based algorithms are suitable for solving problems where the objective function has a smooth profile, and the derivatives of the function are known since they can quickly and efficiently find the local minimum of the objective function. Bee and ant swarm algorithms are better suited for solving problems where the objective function has an irregular profile or only the function values at the mesh nodes are known^5,6^.

Swarm algorithms used in the article are more robust to errors (such as input data errors and algorithm implementation errors) relative to gradient-based algorithms. In addition, ACO and ABC algorithms are more scalable than gradient-based algorithms. Gradient-based algorithms can be difficult to apply to complex problems because they require calculating gradients for all optimized variables.

Swarm algorithms are more efficient than gradient methods because all individuals in the population can solve the problem simultaneously, while gradient-based algorithms can be time-consuming for large problems^7^.

Ultimately, the choice of algorithm depends on the specific problem to be solved. If the problem has many local minima, bee and ant swarm algorithms are better than gradient methods. If the problem has a smooth profile and is easy to solve, then gradient-based algorithms are a better choice.

Local minimums resilience refers to the ability of an optimization algorithm to avoid local minimums that are not global. Gradient optimization algorithms are susceptible to local minimums because they track the directions of the greatest decrease in the objective function, which means that these algorithms will move in the direction in which the value of the objective function decreases the fastest. If the solution space has multiple local minima, the gradient optimization algorithm may get stuck in one of them, skipping the global minimum. ABC and ACO algorithms are less prone to local minimums because they do not rely on differentiation of the objective function, which means these algorithms randomly explore the solution space, increasing the chances of finding a global minimum. However, robustness to local minimums is one of many factors to consider when choosing an optimization algorithm. Other important factors include the algorithm’s complexity and run time. Note, however, that not all inverse problems are the same, and in some cases, gradient optimization algorithms may be a better option^8^.

Both swarm algorithms (ABC and ACO) and gradient methods solve optimization problems, but the first is evolutional, and the second one is analytical. ABC and ACO algorithms are more effective than gradient methods in tackling high-complex optimization problems due to their ability to explore the solution space. Furthermore, gradient approaches can become stuck in local minima, whereas ABC and ACO algorithms can get around this problem. The time consumption of bee and ant swarm algorithms depends on many factors, such as the size of the problem, the number of individuals in the swarm, and the values of the input parameters controlling the algorithm. In general, bee and ant swarm algorithms are considered linear complexity algorithms. Gradient-based algorithms are decision algorithms that guarantee to find the optimized problem’s global minimum. However, they can be time-consuming, especially for large problems (be careful when translating). As with swarm algorithms, the time consumption of gradient algorithms depends on factors such as the size of the problem or the number of variables in the problem. In general, gradient algorithms are considered algorithms of quadratic complexity. On the other hand, ABC and ACO algorithms tend to be slower than gradient methods. This is because ABC and ACO algorithms have to explore the solution space randomly, while gradient methods can quickly use information about the objective function to find a solution. In conclusion, swarm algorithms are considered more efficient but generally slower than gradient methods in solving optimization problems of high complexity^8^.

Finding the shortest path in a city between two points spaced at a given distance from each other is a different problem. Ant Colony Optimization (ACO) algorithm was used to solve it. This algorithm makes full use of the capabilities of distributed and large-scale systems. In a paper^9^, Komar demonstrated a better ACO efficiency than standard navigation to find the shortest route between two points.

In their research, Hetmaniok et al.^10,11^ applied selected swarm intelligence algorithms to address the inverse heat conduction problem, focusing on the third kind of boundary condition. They reconstructed the temperature distribution within the considered area and identified the thermal conductivity parameter as part of the problem-solving process. The accuracy of the approximate solution was assessed by minimizing the functional in the heat conduction problem. The authors emphasized the efficacy of swarm algorithms in solving inverse problems, particularly when considering input errors and parameter selection.

The finite elements method (FEM) for numerical calculations of many phenomena in computer simulations is the most widely used method, for example, in the continuous casting of steel and many others^12–14^. Therefore, the authors use FEM in the numerical part of their study.

Conductivity-radiation transient problems are often encountered in engineering applications such as thermal insulation design and heat transfer analysis in combustion chambers. Many unknown parameters, such as thermal conductivity, emissivity, and absorption, typically characterize these problems. Inverse analysis is the process of estimating unknown parameters from experimental data.

The authors in^15^ present a new inverse analysis method for conductivity-radiation transient problems. The method proposed is based on the Boltzmann method on a grid (LBM) and the finite volume method (FVM). The LBM method is a numerical method for solving transport equations, such as the thermal conductivity equation. The FVM method is a numerical method for solving partial differential equations, such as the radiation equation. The Genetic Algorithm (GA), a stochastic optimization algorithm, was also used to find optimal values for unknown parameters. The authors demonstrated the applicability of artificial intelligence to engineering problems.

The thermal properties of materials are often difficult to measure accurately, especially for materials with variable thermal conductivity, such as composites and porous materials. Inverse analysis is a powerful tool that can be used to recover the thermal properties of materials from experimental data.

The authors in^16^ present a new inverse analysis method for recovering the thermal properties of materials in transient conductivity-radiation problems. The presented method is based on the finite element (FEM), a powerful numerical method for solving partial differential equations. The authors also used the genetic algorithm (GA), a stochastic optimization algorithm, to search for optimal values of thermal properties. It was shown that the presented method can accurately estimate unknown parameters from experimental data.

Fourier’s law of thermal conductivity is a commonly used model for heat transfer in solids. However, there are many situations in which Fourier’s law is not valid, such as in materials with large temperature gradients or materials with inhomogeneous properties. In such cases, it is necessary to use a more general heat transfer model, such as Non-Fourier’s law. In the article^17^, the authors present an inverse analysis method for parameter estimation in non-Fourier’s law. The method presented is based on the finite element method (FEM), a powerful numerical method for solving partial differential equations. For calculations, the authors also used the genetic algorithm (GA), a stochastic optimization algorithm, to find optimal parameter values. The authors demonstrated the effectiveness of the method. The authors apply it to the two-dimensional non-Fourier problem of conductivity and radiation. They showed that the method can accurately estimate parameters in the non-Fourier law from experimental data.

AI algorithms and their applications in areas such as heat conduction showcase the potential for innovative solutions in various fields. The seamless integration of AI methodologies, including swarm algorithms, with mathematical models in heat transfer and other domains will continue to drive progress and open new avenues for research and development.

The conducted research aimed to reproduce the value of the $\kappa$ coefficient that it can take during the solidification of the cast in the casting mold. While the measurement of temperatures in the cast and the casting mold is accurate, the experimental determination of the $\kappa$ coefficient is difficult due to the variability of the gap between the cast and the casting mold. Two swarm algorithms were used for this research. The coefficient $\kappa$ was determined based on temperature changes over time. In addition, to take measurement uncertainty into account in the experiment, it was assumed that temperatures could be read with 0$\%$, 1$\%$, 2$\%$, or 5$\%$ disturbance. The research was tested by conducting a numerical experiment for parameters corresponding to the cooling of an aluminum alloy cast in the casting mold. It is worth noting that there are already publications related to applying artificial intelligence algorithms to casting processes. Some studies have used genetic algorithms; others have used neural networks or other optimization methods. However, our study brings a new perspective to the problem of reproducing casting conditions by focusing on swarm algorithms^10,11^. Our approach shows that swarm algorithms can reconstruct the $\kappa$ coefficient in a computationally efficient and easy-to-implement manner. These algorithms can also be used to reconstruct the value of the heat transfer coefficient occurring in the continuity boundary condition when modeling the thermal conductivity of binary alloys. Other publications have yet to describe this consistent with our state of the art. In addition, the presented aluminum alloy results may be applied in Industry 4.0. Our work can provide valuable guidance to other researchers and practitioners interested in using artificial intelligence to model and simulate casting processes.

In the subsequent section, the authors explore the influence of parameters such as finite element mesh density and input parameters on computer simulations. This analysis is conducted within the context of ABC and ACO calculations as they relate to the reconstruction of the heat conduction coefficient between the cast and the casting mold. The outcomes of these calculations were then compared across different iterations, two distinct tessellations, and a varying number of individuals. The findings present interactions between AI methodologies and mathematical models in heat transfer.

## Mathematical model

### Heat transfer

Transient heat conduction occurs during heating and cooling bodies as long as they strive to achieve temperature equilibrium with the environment in which they find themselves. The heat transfer between the parts of bodies in direct contact with each other is defined as heat conduction. The following formula defines the mathematical model of conduction in a single body $\Omega$:

1 $$\begin{aligned} \rho c\frac{\partial T}{\partial t}+\nabla \cdot (-\lambda \nabla T) = Q, \end{aligned}$$

where $\rho$–material’s density $[\frac{\textrm{kg}}{\textrm{m}^{3}}]$, *Q*–the capacity of internal heat sources $[\frac{\textrm{W}}{\textrm{m}^{3}}]$ (in this paper $Q = 0$ due to lack of such sources), $\nabla$–differential nabla operator, *T*–temperature [K], *c*–specific heat $[\frac{\textrm{J}}{\textrm{kg K}}]$, and $\frac{\partial T}{\partial t}$ is the time derivative of temperature. The considered issue is one of the initial-boundary problems, therefore requiring appropriate initial and boundary conditions. As the initial conditions, authors used the Cauchy conditions that assign specific temperature values at the initial instant $t_ {0}$ = 0 [s] what is necessary to determine the temperature distribution^18^:

2 $$\begin{aligned} T({\textbf{r}},t)|_{t=0}=T_{0}({\textbf{r}}), \end{aligned}$$

where ${\textbf{r}}$ is the field vector at a given point.

Boundary conditions are essential for solving the heat transfer equation. With a suitable set of them, the solution can be clearly determined. The chosen type of boundary condition depends on the specific problem. In some cases, only one type of boundary condition can be used. In other cases, using more than one type of boundary condition may be necessary. We distinguish four types of boundary conditions associated with heat transfer:

One of the boundary conditions in the casting process is the Dirichlet condition. In this case, the temperature at the surface of the casting is known.

Boundary condition of the first type (Dirichlet)—on the boundary $\Gamma _A$ of area $\Omega$ the temperature distribution is given ($T_{z}$)

3 $$\begin{aligned} \Gamma _A:T = T_{z}, \end{aligned}$$

The Neumann condition is used when the heat flux at the surface of the casting is known. The natural condition is used when the heat flux at the surface of the casting is 0.

Boundary condition of the second type (von Neumann)—on the boundary $\Gamma _B$ of area $\Omega$, the heat flux is known ($q_{z}$)

4 $$\begin{aligned} \Gamma _B:q=(q_{z}), \end{aligned}$$

Robin’s condition or Newton’s cooling law is used when the temperature at the surface of the casting and the heat transfer coefficient with the surroundings are known.

Boundary condition of the third type (Newton’s or Robin’s)—on the boundary $\Gamma _C$ of area $\Omega$ heat exchange with the environment takes place:

5 $$\begin{aligned} \Gamma _C:q=\alpha (T-T_{env}), \end{aligned}$$

where $\alpha$ is the heat transfer coefficient of exchange with the environment, *T* is the temperature at the boundary between the body and $\Gamma _C$, $T_{env}$ is the ambient temperature, *q* denotes the heat flux inputting ($T <T_{env}$ ) into the area $\Omega$ or outputting ($T >T_{env}$ ) from the area $\Omega$,

The continuity boundary condition (the fourth kind) is used when heat flows between two areas (e.g., the casting and the casting mold). It can be applied in two cases: ideal contact or through a layer separating the cast and the casting mold (e.g., an air gap). The boundary conditions significantly affect the temperature distribution in the casting, so they must be carefully selected to ensure that the desired properties of the cast are achieved.

Boundary condition of the fourth type (continuity condition)—on the boundary $\Gamma _D$ separating areas $\Omega _{1}$ and $\Omega _{2}$ heat exchange occurs. Two cases are possible here:

- ideal contact between areas,
- lack of ideal contact—heat exchange through the separation layer describe $\kappa$ coefficient:
  6 $$\begin{aligned} \kappa = \frac{\lambda _{p}}{\delta }, \end{aligned}$$
  where $\lambda _{p}$ is the thermal conductivity coefficient of the separation layer, and $\delta$ is the thickness of that layer^18,19^.

The numerical case described in the article^20^ presents a more complex casting model; the model presented in the reviewed article is much simpler. In the current article, the model does not consider mechanical interactions and uses simplified geometry. However, it is free to move on to realistic problems, as the swarm algorithms used do not impose any restrictions on the numerical model:

- modeling casting of metal alloys—in this case, many factors must be taken into account, such as the temperature of the alloy, the temperature of the mold, the mechanical properties of the alloy and the mold, and the solidification speed of the alloy,
- modeling the hardening process of steel—in this case, also needs to consider many factors, such as the temperature of the steel, the temperature of the quenching oil, the cooling rate of the steel, and the mechanical properties of the steel.

The proposed optimization methods with ABC and ACO algorithms can successfully solve these problems with high accuracy.

#### Artificial intelligence algorithms

The ABC and ACO algorithms are classified as swarming algorithms and belong to metaheuristics algorithms. A metaheuristic is a general computational problem-solving method used to solve any problem described by the terms defined by this algorithm. They are often based on analogies to real-world processes (physics, chemistry, biology) that can be interpreted in terms of optimization^21^. Metaheuristics make obtaining results close to the optimum possible without requiring expert knowledge of a specific optimization problem. These algorithms quickly adapt to constraints and the solution space size while not depending on the number of variables. The authors, B. Gerardo et al., in the paper^22^ and S. Hackwood, in the paper^2^, presented the concept of swarm intelligence. The inspiration for the development of these algorithms came from observations of biological systems such as herds of birds, swarms of ants, colonies of worms, or just swarms of bees.

The artificial bee and ant colony are two of the most popular swarm-based optimization algorithms. They are easy to understand and implement and perform well in many optimization problems. There are many other swarm-based optimization algorithms (e.g., particle swarm, beetle swarm), but these algorithms are more complex and harder to implement.

Additional reasons why ABC and ACO were chosen are:

- scalability to large problems,
- resistance to local minimum narrowing,
- ease of adaptation to other problems,
- ease of implementation in various programming languages.

In general, bee and ant artificial colony algorithms are powerful tools for solving optimization problems, are easy to understand and implement, and have outstanding computational performance.

#### Bee algorithm

The artificial bee colony that makes up the ABC algorithm comprises two groups of bees. The first half of the colony consists of worker bees. The second half of the colony comprises equal parts of non-employed bees.

One of the assumptions of the bee algorithm is that the number of non-employed bees is equal to employed bees. It means only one bee belongs to one food source in a given surround. If a food source explored by employed bees becomes exhausted, such bees become unemployable.

A possible solution of the problem in the ABC algorithm is the positions of the food sources. The amount of nectar present in the food source determines the quality of the solution (efficiency). The first step of the ABC algorithm is randomly generating the initial *P* population (number of food sources) and *SN* solutions (the number of the explored food sources).

Each time a solution $x_{i}$ $(i = 1, 2,\ldots , SN)$ is identical to the position of the food source. Iterations are responsible for updating the solution, where the determination of the coordinates of the source position after initialization is subjected to multiple transitions.

Information is exchanged between the worker bees and the non-employed bees. Updating the change in solution by the employed bee occurs depending on local information, and the new source is tested depending on the amount of nectar. The new position of the food source is remembered only if the amount of nectar in the next iterations exceeds the value of the previous iterations. Otherwise, the previous position is remembered. Employed bees, after the foraging process, share information about the amount of nectar, which is calculated from the formula:

7 $$\begin{aligned} fit_{i}={\left\{ \begin{array}{ll} \frac{1}{1+J(x_{i})}&{} \text {if}J(x_{i}) \ge 0,\\ {1+|J(x_{i})|}&{} \text {if}J(x_{i}) < 0. \end{array}\right. } \end{aligned}$$

where $J(x_{i})$ determines the quality of a given source $x_i$. The bees’ choice of food source depends on the amount of nectar (${fit_{j}}$) found in the food source.

The probability value $p_{i}$ of choosing a food source is the primary criterion for selection by an unemployed artificial bee and is calculated according to the formula^4,23^:

8 $$\begin{aligned} p_{i}=\frac{fit_{i}}{\sum \nolimits _{j=1}^{SN}fit_{j}}, \;\;i=1,\ldots , SN, \end{aligned}$$

Afterwards, the updating of food source coordinates $v_{ij}$ comes according to the relation:

9 $$\begin{aligned} v_{i}^{j}=x_{i}^{j}+\phi _{ij}(x_{i}^{j}-x_{k}^{j}), \end{aligned}$$

where $k \in \{1, 2,\ldots , SN\}$ and $\phi _{ij}$ $\in [-1, 1]$ is also a random number and $j \in \{1, 2,\ldots , D\}$. A dimensional vector of $x_{i}$ is the *D* parameter and denotes the number of optimization parameters in the ABC algorithm. The variable *k* must be different from *i*.

#### Ant algorithm

The ACO algorithm is used when considering problems of finding the shortest path in a graph and is inspired by real ants. The search is done by finding the shortest path between the anthill and the food. The ants randomly choose the food direction, leaving a pheromone passage on their way back to the anthill. This passage left on a specific path gradually evaporates if other ants do not frequent the path. Ants are more likely to choose shorter routes because, on such routes, the pheromone passage steams much more slowly than on a longer path. It is interesting to note that when ants discover a better path, more of them will start using it, which is known as the positive feedback phenomenon.

The artificial ants cooperate in searching for the most favorable solution to difficult combinatorial problems. In the search for this solution, there is a relationship between the ants and the accumulated experience they use. Ants work out a common set of solutions over time in the form of shortest paths that lead to their goal. However, there are differences between artificial and real ants. Artificial ants move within the input graph’s edges, while ants can choose any route in nature. The quality of the solution in the ACO algorithm is related to the pheromone passage. One characteristic of an artificial ant colony is that each ant finds the expected solution in each passage. As a result of the algorithm, the best solution is obtained, which is found by the best ant. The pheromone passage is updated along the route when the route found by the artificial ant turns out to be better than the one generated so far. Through this, subsequent ants choose certain edges in the graph more willingly. The trace-reinforced ants’ process is influenced by the distance of the anthill from the foraging area (the path length in the graph). There is a higher probability that the next ant will follow the trail of its predecessor if the pheromone passage is stronger.

The passage routes of all ants follow the rules: at the beginning, the nodes across which the ant will pass are determined at random $k \; (k=1, \ldots , M)$, *M*—a number of ants. The probability $p_{ij}$ of choosing *j*th node by the ant being in *i*th node is defined by the equation:

10 $$\begin{aligned} p_{ij}^{k}(t) = \frac{[\tau _{ij}(t)]^{\alpha }[\eta _{ij}(t)]^{\beta }}{{{\sum \limits _{i,j{\in }G}}}[\tau _{ij}(t)]^{\alpha }[\eta _{ij}(t)]^{\beta }}, \quad i=1, \dots , D, \quad j=1, \ldots , R, \end{aligned}$$

where $\eta$—is the heuristic function, $\alpha$ and $\beta$ are constants that determine the effect of pheromone values and heuristic values on the *k*th ant’s decision, *G* is a path in the graph traversable by *k*th ants, $\tau _{ij}$ and $\tau \in (0, 1\rangle$ the pheromone array in which the information about the amount of pheromone left is stored, *t*—time step iteration, *R*—node in the graph.

The best way to remember a path is when the new one is better than the previous one. When all ants have passed through all paths, then the pheromone array is updated under the formula:

11 $$\begin{aligned} \tau _{ij}(t+1)=(1 - \rho )\tau _{ij}(t)+{\sum _{k=1}^{M}} \Delta \tau _{ij}^{k}(t)+\rho \Delta \tau _{ij}^{best}(t), \end{aligned}$$

where values $\Delta \tau _{ij}^{k}$ is the amount of pheromone left by the *k*th ant on the movement path, $\Delta \tau _{ij}^{best}$ is the amount of pheromone left by the best ant on the path of movement, $\rho$ is the evaporation coefficient in the range $( 0, 1\rangle$, which determines what part of the pheromone is to remain (0—evaporates everything, 1—nothing evaporates).

During the algorithm operation, a pheromone steaming is added to preserve against the unlimited growth of the pheromone passage. The roulette wheel method randomizes nodes on the paths for each ant during the first iterations. The probability calculated from the equation (Eq. 10) is considered during this random selection. The path characterized by the best quality index is found on the path from the anthill to the feeding ground after the first pass of all ants. The path transition for an ant is modified after the definition of the quality indicator in case it obtained the highest rating. In each layer of the graph, new nodes are determined at random for the path of passage that is the best. The path nodes approximate the nodes in each layer in case they have the best quality index. Then, given the formula (Eq. 11), each calculation iterations’ pheromone array is modified. Once this is done, the probability calculation is engaged, taking into account the determined pheromone array $\tau$, and proceeded to the next iterations of calculation^24,25^.

## Assumptions of the research

The paper deals with an issue that needs an association of two different subject areas: thermomechanics (heat conduction) and computer science (artificial intelligence algorithms modeled on nature). The analyzed physical heat conduction processes considered the continuity boundary condition (of the fourth type). The obtained results were tested regarding the influence of input parameters in swarming algorithms on computer simulations of heat conduction. The work concerned the value reconstruction of the $\kappa$ coefficient occurring in the non-ideal contact with the separating layer. For this purpose, two nature-inspired algorithms were used, i.e., artificial bee (ABC) and ant (ACO) colonies.

The parametric settings associated with the ABC and ACO algorithms were taken from literature items^10,11^. The main parameters in our calculations, respectively, are:

1. for the algorithm ABC:
  food sources—the number of bees in the population,
  search area—the range of the kappa parameter
  a number of iterations—the rerun number of food source searching processes by individuals, consistent with the results presented in the calculations.
2. for the algorithm ACO:
  number of iterations—the rerun number of food source searching processes by individuals, consistent with the results presented in the calculations,
  the initial number of ant population—the smallest number of individuals as a fixed parameter of the algorithm,
  the number of ants in the population—the actual number of individuals as an input parameter of the algorithm,
  Intensification Factor (Selection Pressure)—the value of 0.5, the fixed default parameter of the algorithm,
  Deviation-Distance Ratio—value 1, the fixed default parameter of the algorithm search area—the range of the kappa parameter.

Geometrical model and finite element meshes were created in the GMSH^26^ program. The TalyFEM^27^ library and algorithms implemented in C++ were used in numerical calculations. TalyFEM is a tool that employs the finite element method of the selected physical phenomena simulation. It taps data structures from the PETSc^28^ library, including vectors, matrices, or ready-made solvers.

These libraries were used to implement the numerical model. In^29^, the authors used the same FEM model to simulate the temperature distribution in a DC motor with permanent magnets. The model included a rotor, a stator, and a winding. The authors used various boundary conditions, including constant rotor and stator surface temperatures and heat flow through the bearings. The authors compared their results with those obtained from an experiment on the heat conduction problem. This validation indicates that the FEM model is accurate and can be used to simulate the temperature distribution.

The tests were performed on a computer with the following parameters: Intel (R) Core (TM) i5-4590 CPU @ 3.30 GHz processor, x86_64 architecture, using the Linux operating system in Ubuntu distribution.

Swarming algorithms have been implemented in Python and adapted to the possibility of combining them with the TalyFEM^30^. The error of the approximate solution was minimized using the bee and ant algorithms, respectively. The reference temperature values and the temperatures obtained during the simulation were generated with the constant reference heat transfer coefficient $\kappa$.

The tests used two different finite element mesh density variants for the same geometrical model. Simulations were carried out for one $\kappa$ parameter. It means that coefficient optimization from one range of values ($900{-}1500 \; [\textrm{W}/\textrm{m}^{2}\textrm{K}$]). The reference temperatures were obtained for the reference coefficient of $\kappa = 1000 \; [\textrm{W}/\textrm{m}^2\textrm{K}$]. All simulations were performed for the Al-2$\%$Cu alloy. Material properties are presented in Table 1. The values in Table 1 correspond to the material properties of the frequently used aluminum alloy with the addition of 2$\%$ copper and the material properties of the metal casting mold^18^. The initial temperatures were respectively $T_0 = 960 \; [\textrm{K}$] for cast and $T_0 = 590 \; [\textrm{K}$] for the casting mold.

**Table 1:** Material properties.

	Cast	Casting mold
$\rho$, kg$/\textrm{m}^{3}$	2824	7500
c, J/kgK	1077	620
$\lambda$, W/mK	262	40

2D finite element meshes are described by a full tessellation, including element nodes, interface, surfaces, edges, and vertices sets of the domain. Two meshes of 111 and 576-nodes tessellation are considered in our paper.

For the calculations presented in the article, the number of iterations (the input parameter of the algorithm) was the criterion for finishing the calculations. The convergence control of the algorithm was evaluated on the basis of the value of the functional:

12 $$\begin{aligned} J(\kappa )=\sum _{i=1}^{N_{1}} \sum _{j=1}^{N_{2}}(T_{ij}-U_{ij} )^{2}, \end{aligned}$$

where *i* is the number of nodes in the FEM mesh, *j* is the number of time steps, $N_i$ is the number of nodes in all pairs considered, $N_j$ is the number of time steps, and, $T_{ij}$ is the benchmark temperatures generated at a constant benchmark heat transfer coefficient $\kappa$ and $U_{ij}$ denotes the temperatures obtained during the simulation^31^.

**Figure 1:**
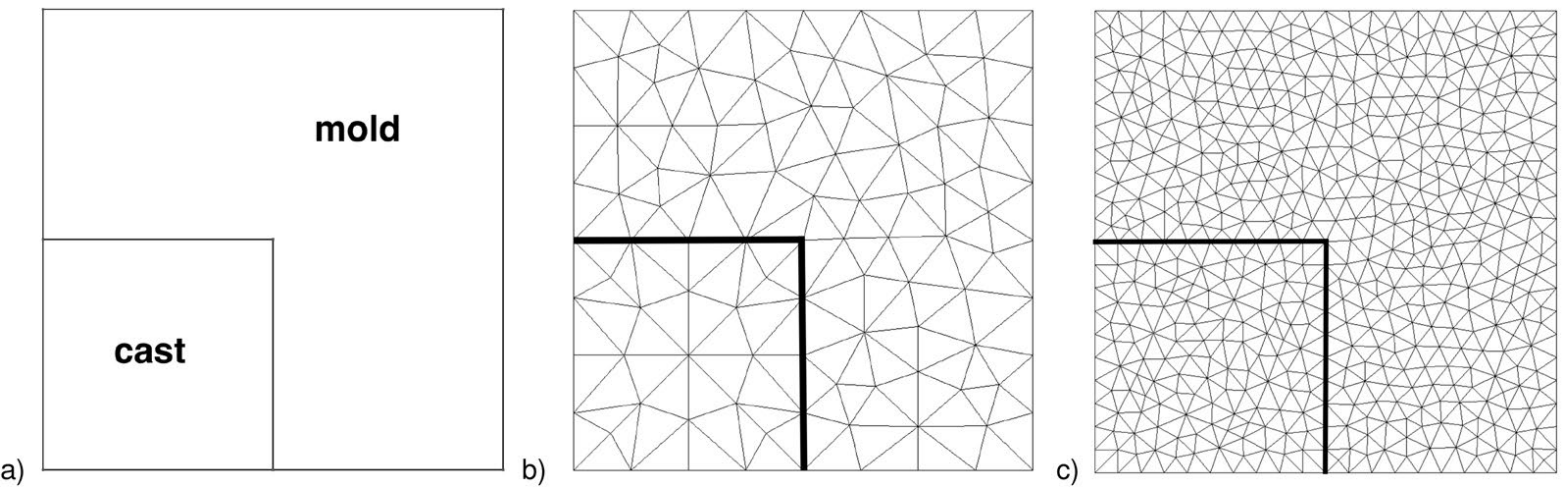
Geometry (**a**) with 111-nodes (**b**) and 576-nodes (**c**) finite element mesh.

The obtained results concern the layer separating the cast and the casting mold for two different tessellations (Fig. 1). The nodes at the interface between the cast and the casting mold have the same spatial coordinates, simplifying the implementation of the boundary condition of the fourth type in the heat conduction model.

Limitations of the ongoing research are related to the need for more time records for the calculations, the somewhat simple design, or the limited hardware resources. However, these limitations are temporary and easily eliminated by exploiting the scalability properties of the algorithms and parallelizing the calculations.

## Results

For each tessellation, calculations were performed for the ABC and ACO algorithms for populations of 15, 17, and 20 individuals and 2 and 6 iterations. In order to decrease the search area and get correct results, a feature that characterizes heuristic algorithms, i.e. the need for multiple runs, was used. For the purpose of the calculations performed in each case, the algorithms were run three times. The 0$\%$, 1$\%$, 2$\%$, and 5$\%$ disturbance of reference values were also included in each case.

In the case of our calculations, disturbances were introduced into the temperature values.

In general, the measurement of temperatures with a thermocouple during the experiment is characterized by good accuracy. It is assumed that, for thermocouples, the measurement accuracy is within $+- 2$ degrees C and the disturbances value not exceeding our assumed 5$\%$. As suggested by experience, we chose four disturbance values from 0 to 5$\%$. At 5$\%$ disturbance, we noticed that the ABC and ACO algorithms’ input parameters do not guarantee finding the best solution relative to the reference value^32^.

In our research, we used a uniform distribution for disturbance represented by the random.uniform() function available in Python. The range of disturbance is symmetric, meaning that for a 5$\%$ disturbance in temperature values, the range was $-2.5$ to $+2.5\%$.

**Figure 2:**
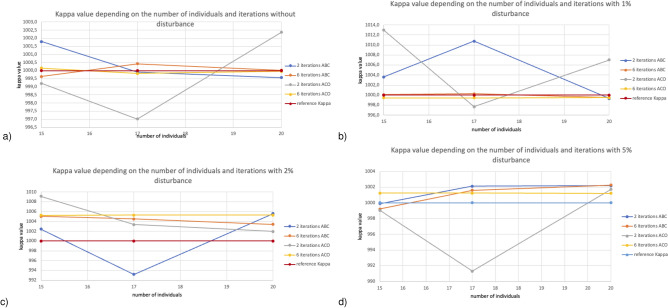
Kappa value depends on the number of individuals, iteration, and interference for finite element mesh with 111 nodes.

Figure 2 presents the reconstructed value $\kappa$ depending on the number of individuals (for 15, 17, and 20) and iteration (2 and 6) for a finite element mesh with 111 nodes. Figure 2a at 0$\%$ disturbances in reference values for 15, 17, and 20 individuals, the ABC algorithm proved slightly better. In contrast, for 20 individuals, the best results in relation to reference values are obtained for six iterations for both algorithms. Figure 2b at 1$\%$ disturbance of 6 iteration reference values for both algorithms, regardless of the number of individuals, they selected the value of the $\kappa$ parameter at a satisfactory level. Figure 2c at 2$\%$ disturbances in reference values for 15 individuals, the ABC algorithm obtains the best values at two iterations. In contrast, ACO obtains the best values for 17 and 20 individuals with two iterations. Both algorithms obtain comparable results. Figure 2d at 5$\%$ disturbances in reference values shows that the ABC algorithm with two iterations for 15 individuals proved slightly better than ACO. When considering 17 individuals, both algorithms for six iterations obtained similar values. The ACO algorithm for 2 and 6 iterations better chose the $\kappa$ parameter for 20 individuals. Summarizing at 5$\%$ disturbance of reference values, ABC and ACO algorithms for six iterations obtained the best results.

**Figure 3:**
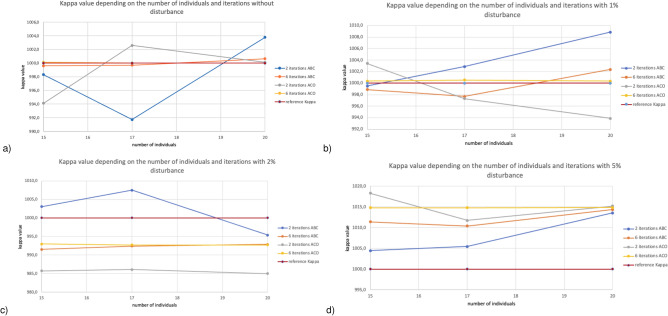
Kappa value depends on the number of individuals, iteration, and interference for finite element mesh with 576 nodes.

Figure 3 presents the reconstructed value $\kappa$ depending on the number of individuals (for 15, 17, and 20) and iteration (2 and 6) for a finite element mesh with 576 nodes. Figure 3a for 0$\%$ disturbances in reference values show that regardless of the number of individuals, both ABC and ACO for six iterations achieve the best results. Figure 3b for 1$\%$ disturbances in reference values regardless of the number of individuals, the best results are obtained for six iterations of the ACO algorithm. However, the results for ABC and ACO for six iterations and 15 and 20 individuals are very similar. ABC obtains the values closest to the reference values in Fig. 3c at 2$\%$ disturbance after two iterations regardless of the number of individuals. Figure 3d at 5$\%$ disturbances of reference values for 15 and 17 individuals, the ABC algorithm is already similar to the reference value at two iterations. When considering results for 20 individuals, both ABC and ACO obtain similar results.

**Figure 4:**
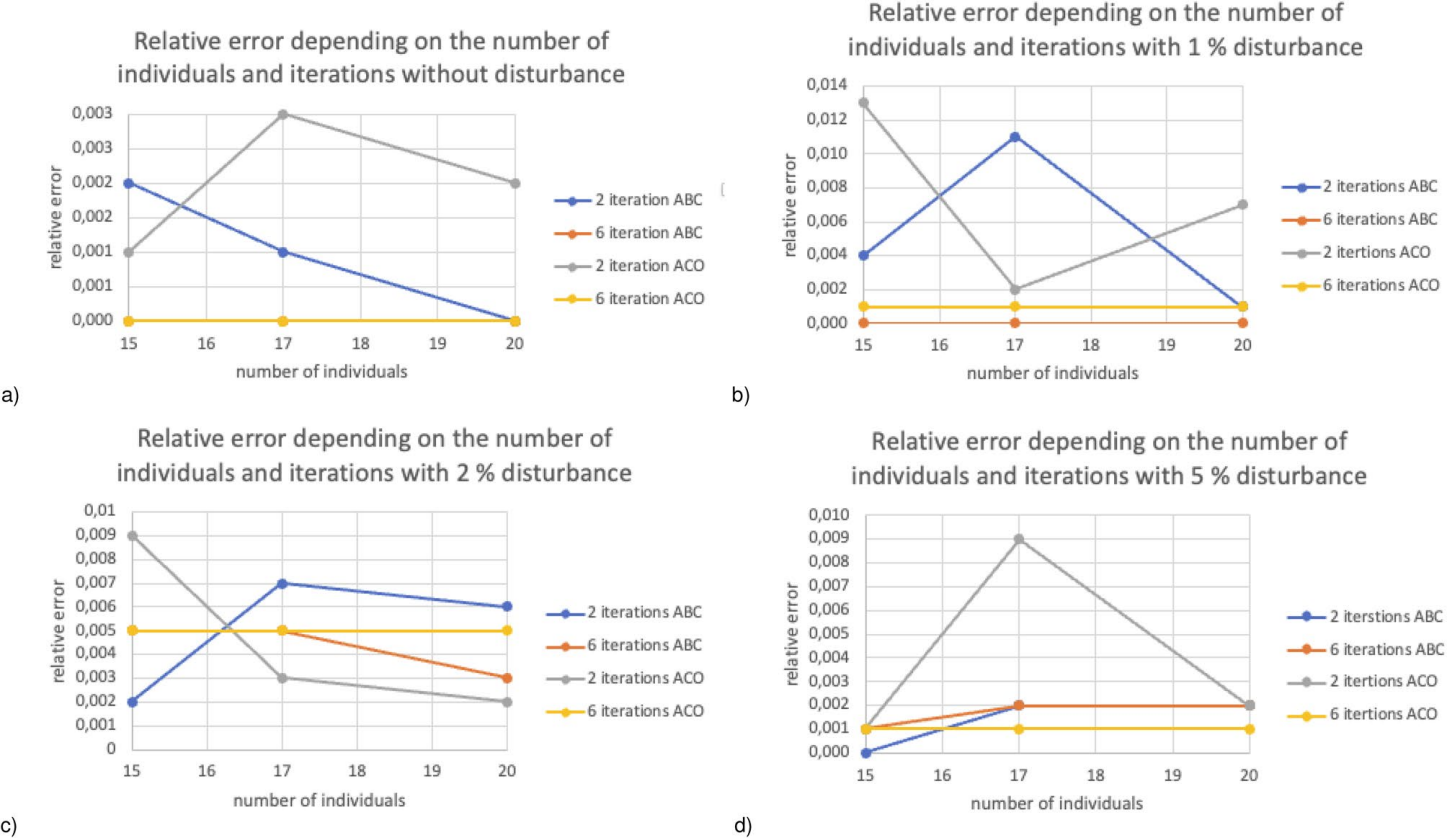
Relative error depending on the number of individuals and iterations for finite element mesh with 111 nodes.

Figure 4 presents relative error depending on the number of individuals (for 15, 17, and 20) and iteration (2 and 6) for finite element mesh with 111 nodes. Figure 4a for 15 and 20 individuals, the error values did not exceed 0.004$\%$. The smallest error was generated at 0$\%$ disturbance of six iteration reference values for both algorithms. Figure 4b regardless of the number of individuals, the ABC algorithm for six iterations showed the smallest error in relation to the reference values. However, for two iterations it was an ACO algorithm. Figure 4c the smallest error with two iterations was obtained for ABC with 15 individuals and 20 individuals for ACO and did not exceed 0.006$\%$. Both algorithms obtain a similar error in the calculation for 17 individuals for six iterations. Figure 4d the ABC algorithm already with two iterations for 15 individuals selects values so that the error does not exceed 0.002$\%$. ACO for 17 and 20 individuals with six iterations obtained an error not exceeding 0.002$\%$.

**Figure 5:**
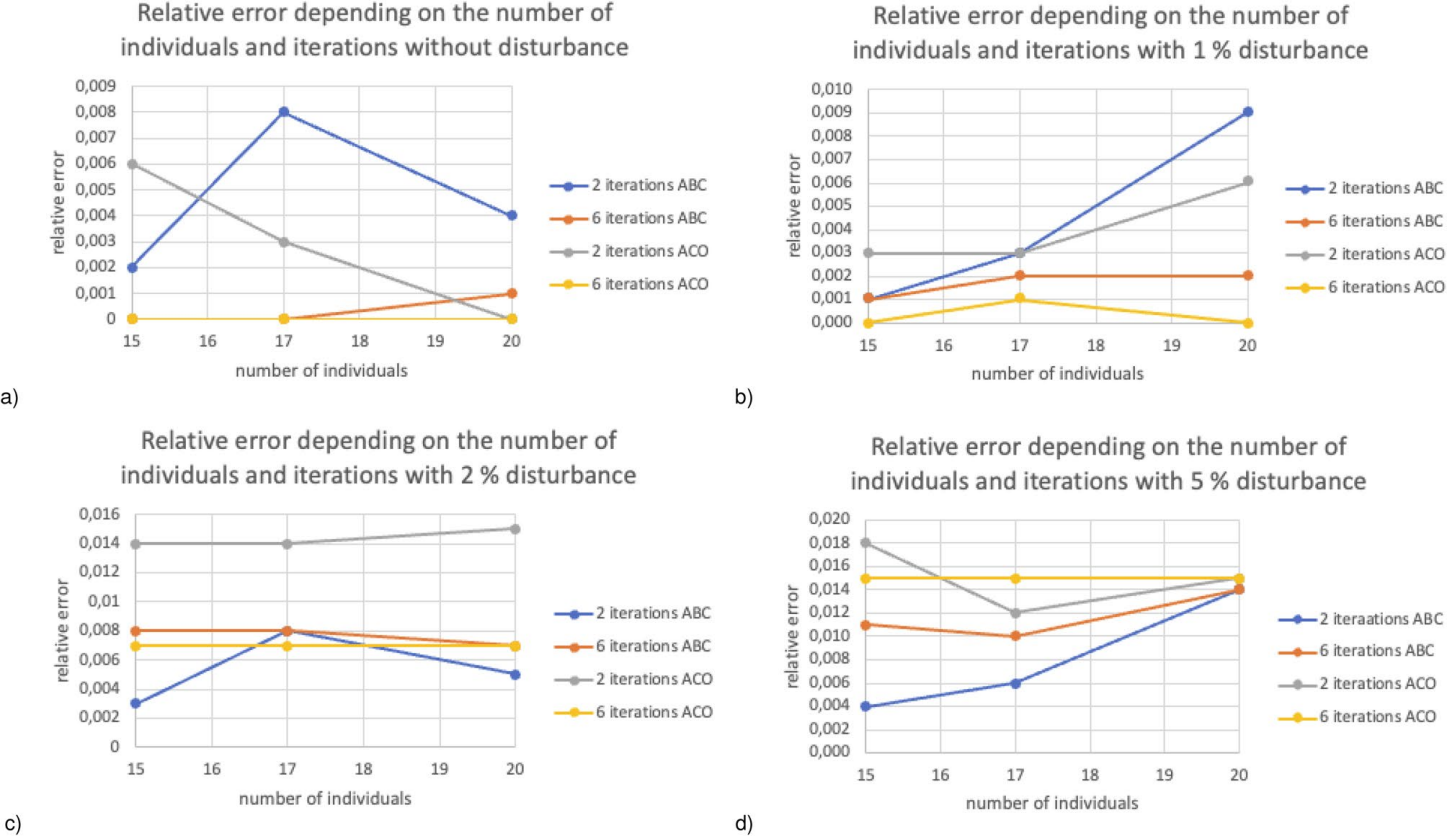
Relative error depending on the number of individuals and iterations for finite element mesh with 576 nodes.

Figure 5 presents relative error depending on the number of individuals (for 15, 17, and 20) and iteration (2 and 6) for finite element mesh with 576 nodes. Figure 5a taking into account 0$\%$ disturbances values of both ABC and ACO give satisfactory results for six iterations regardless of the number of individuals, and the relative error did not exceed 0.001$\%$. In contrast, the results obtained for two iterations are more susceptible to changes in the number of individuals regardless of the selected algorithm. Figure 5b with two iterations, increasing the number of individuals causes a more significant relative error. However, increasing the number of individuals for six iterations does not significantly affect a relative error that remains unchanged or slightly decreases. Figure 5c the ABC algorithm for two iterations taking into account 15 and 20 individuals obtained an error not exceeding 0.005$\%$. Six iterations for both algorithms obtained an error oscillating the values of 0.008$\%$, regardless of the number of individuals. The results obtained using the ABC and ACO algorithms are very similar regardless of the number of individuals, and the relative error oscillates around 0.007$\%$. Figure 5d the ABC algorithm shows a smaller relative error regardless of the number of iterations and the number of individuals. However, the results obtained for 17 individuals have the least relative error in each case.

Table 2 involves calculations for the bee and ant algorithm for 15 individuals and a grid of 111 and 576 nodes, respectively, and table 3 shows the relative error for the reconstructed $\kappa$ coefficient, taking into account two different tessellations and ABC and ACO.

Authors picked up that better, in most cases, results come from using the ABC algorithm for the finite element mesh of 111 nodes. For the 1$\%$, 2$\%$, and 5$\%$ disturbances, the results obtained with this algorithm have values closer to the expected $\kappa$ factor. On the other hand, the ACO algorithm improved for the 0$\%$ disturbance of reference temperatures. Six iterations were sufficient for a minor disturbance (0$\%$, 1$\%$) to obtain satisfactory values of the $\kappa$ coefficient. On the other hand, for a more considerable disturbance (2$\%$, 5$\%$), both algorithms needed only two iterations to obtain results at a satisfactory level relative to the reference values of the $\kappa$ coefficient. Finally, the differences in the obtained values of the $\kappa$ coefficient between ABC and ACO needed to be more significant to use both algorithms for calculations with equal success.

For a finite element mesh divided into 576 nodes, generally better results are obtained with ACO than with ABC. Only for two iterations with 2$\%$ disturbances in the temperature reference values did the ABC algorithm obtain better values for the $\kappa$ parameter. For this finite element mesh density, the differences between the expected values obtained by the two algorithms are insignificant.

For a 15 individuals population, the best results are obtained by the ABC algorithm for the tessellation of 111 nodes. In contrast, for the tessellation of 576 nodes, the ACO algorithm proved to be better. The value of the coefficient *kappa* does not differ from the expected value of more than 0.09$\%$ (Table 3). Nevertheless, the values of the relative error are so small that it can be concluded that both algorithms select the value of the coefficient of $\kappa$ not differing from the reference value by more than 0.02$\%$.

**Table 2:** Reconstructed value of the coefficient $\kappa$ for 15 individuals using ABC and ACO algorithms.

Noise	Iterations	$\kappa$ (111 nodes)		$\kappa$ (567 nodes)	
		ABC	ACO	ABC	ACO
$0\%$	2	1001.794	999.211	998.316	994.122
	6	999.640	1000.162	999.598	1000.13
$1\%$	2	1003.622	1012.984	999.497	1003.413
	6	1000.079	999.469	998.869	1000.374
$2\%$	2	1002.426	1009.13	1003.033	985.741
	6	1005.037	1005.282	991.501	992.997
$5\%$	2	999.890	999.042	1004.475	1018,302
	6	999.225	1001,234	1011.439	1014.843

**Table 3:** Relative error for 15 individuals using ABC and ACO algorithms.

Noise	Iterations	$\delta$ (111 nodes)		$\delta$ (567 nodes)	
		ABC	ACO	ABC	ACO
$0\%$	2	0.002	0.001	0.002	0.006
	6	0.000	0.000	0.000	0.000
$1\%$	2	0.004	0.013	0.001	0.003
	6	0.000	0.001	0.001	0.000
$2\%$	2	0.002	0.009	0.003	0.014
	6	0.005	0.005	0.008	0.007
$5\%$	2	0.000	0.001	0.004	0.018
	6	0.001	0.001	0.011	0,015

Table 4 presented calculations for the ABC and ACO algorithm for 17 individuals and a grid of 111 and 576 nodes, respectively, and table 5 shows the relative error for the reconstructed $\kappa$ coefficient, taking into account two different tessellations and ABC and ACO.

For a tessellation equal to 111 nodes at 0$\%$ and 5$\%$, disturbance of the ACO algorithm was better and needed six iterations to obtain results close to the expected value of the $\kappa$ coefficient. In contrast, for the 2$\%$ disturbance, only two iterations were needed. For a 2$\%$ disturbance, this tessellation needed only two iterations, for which the error determining the difference between the expected value and the obtained value did not exceed 0.003$\%$. The ABC algorithm only for the 1$\%$ disturbance was better than the ACO algorithm, and it needed six iterations to obtain results with a relative error of no more than 0.0003$\%$.

The presented results show that for a finite element mesh divided into 576 nodes at 0$\%$, 1$\%$, 2$\%$ disturbance, the ACO algorithm proved to be slightly better than the ABC algorithm for six iterations. In this case, the relative error does not exceed 0.013$\%$. On the other hand, at 5$\%$ disturbance, the ABC algorithm proved to be better, with the help of which fair values were obtained in 2 iterations.

The results of numerical simulations obtained for 17 individuals using ABC and ACO give almost identical results, as evidenced by the tiny or zero relative error. The values obtained for the two algorithms differ slightly from the expected value of the $\kappa$ coefficient, and the error does not exceed more than 0.013$\%$ (Table 5).

**Table 4:** Reconstructed value of the coefficient $\kappa$ for 17 individuals using ABC and ACO algorithms.

Noise	Iterations	$\kappa$ (111 nodes)		$\kappa$ (567 nodes)	
		ABC	ACO	ABC	ACO
$0\%$	2	999.915	997.014	991.737	1002.602
	6	1000.429	999.821	999.698	1000.029
$1\%$	2	1010.785	997.702	1002.894	997.281
	6	1000.282	999.440	997.684	1000.556
$2\%$	2	993.197	1003.360	1007.46	986.098
	6	1004.494	1005.314	992.395	992.700
$5\%$	2	1002.114	991.296	1005.455	1011.746
	6	1001.607	1001.249	1010.391	1014.796

**Table 5:** Relative error for 17 individuals using ABC and ACO algorithms.

Noise	Iterations	$\delta$ (111 nodes)		$\delta$ (576 nodes)	
		ABC	ACO	ABC	ACO
$0\%$	2	0.001	0.003	0.008	0.003
	6	0.000	0.000	0.000	0.000
$1\%$	2	0.011	0.002	0.003	0.003
	6	0.000	0.001	0.002	0.001
$2\%$	2	0.007	0.003	0.008	0.014
	6	0.005	0.005	0.008	0.007
$5\%$	2	0.002	0.009	0.006	0.012
	6	0.002	0.001	0.010	0.015

Table 6 presented calculations for the ABC and ACO algorithm for 20 individuals and a grid of 111 and 576 nodes, respectively, and Table 7 shows the relative error for the reconstructed $\kappa$ coefficient, taking into account two different tessellations and ABC and ACO.

The results presented for 111 nodes in the finite element mesh showed that ACO at 0$\%$, 2$\%$, 5$\%$ disturbance obtains slightly better results than ABC. For the more considerable disturbance (2$\%$, 5$\%$), the ACO algorithm needed only two iterations to get a value close to the expected value. In contrast, in the other cases, six iterations were needed regardless of the type of swarm algorithm. The relative error value in each case did not exceed 0.003$\%$.

For the tessellation of 576 nodes, the ACO algorithm also proved slightly better. However, in this case, the results obtained had the same trends as previous calculations for a smaller number of individuals in the population. Correspondingly, the results closest to the expected value were obtained for 0$\%$ and 1$\%$ disturbance and six iterations by the ACO algorithm, for 2$\%$ disturbance and six iterations by the ABC algorithm, and 5$\%$ disturbance and two iterations by the ACO algorithm.

During the calculations, both algorithms obtain results for both tessellations very close to each other and at a satisfactory level. In neither case, even in the worst case, the relative error between the expected and obtained values exceeded 0.015$\%$.

**Table 6:** Reconstructed value of the coefficient $\kappa$ for 20 individuals using ABC and ACO algorithms.

Noise	Iterations	$\kappa$ (111 nodes)		$\kappa$ (567 nodes)	
		ABC	ACO	ABC	ACO
$0\%$	2	999.567	1002.37	1003.785	1000.114
	6	1000.024	999.952	1000.645	1000.006
$1\%$	2	999.318	1007.047	1008.855	993.864
	6	999.512	999.476	1002.378	1000.365
$2\%$	2	1005.618	1001.944	995.367	985.028
	6	1003.392	1005.303	992.904	992.737
$5\%$	2	1002.170	1001.706	1013.558	1015.200
	6	1002.260	1001.214	1014.360	1014.888

**Table 7:** Relative error for 20 individuals using ABC and ACO algorithms.

Noise	Iterations	$\delta$ (111 nodes)		$\delta$ (576 nodes)	
		ABC	ACO	ABC	ACO
$0\%$	2	0.000	0.002	0.004	0.000
	6	0.000	0.000	0.001	0.000
$1\%$	2	0.001	0.007	0.009	0.006
	6	0.000	0.001	0.002	0.000
$2\%$	2	0.006	0.002	0.005	0.015
	6	0.003	0.005	0.007	0.007
$5\%$	2	0.002	0.002	0.014	0.015
	6	0.002	0.001	0.014	0.015

## Conclusions

This paper presented an analysis of input parameters for reconstructing the value of *kappa* heat conduction coefficient in the layer separating the cast and the casting mold. The numerical experiment used bee and ant colony optimization algorithms. The studied parameters were different tessellations, varying numbers of individuals, percentage perturbation of benchmark temperature values, and different the numbers of iterations.

As a result, researchers concluded that all examined parameters’ effect on reconstruction $\kappa$ coefficient value. However, the results obtained by the ABC and ACO algorithms during the calculations are almost identical. The relative errors between the values obtained by the optimization algorithms and the expected value of the coefficient $\kappa$ did not exceed approximately 0.02$\%$ in the worst case.

Generally, it can be said that six iterations in the vast majority of computational cases give a more successful result in reconstructing the coefficient $\kappa$ relative to the reference values. The reconstructed coefficient values never exceed the size of the introduced disturbance of the input parameters. The authors showed similar trends during preliminary research presented in the article^33^.

Although the numerical case described in the reviewed article is somewhat simple, the proposed schemes, due to their scalability, can be used to solve more realistic problems with high accuracy.

This article is a noteworthy contribution to the field of research on the application of swarm algorithms. Swarm algorithms possess the capability to enhance production processes, conduct data analysis, and facilitate data-driven decisions. According to the literature^34^, swarm algorithms have been successfully used to optimize the production planning problem or resource allocation process in a rapidly developing field, and swarm algorithms are one of many digital technologies that can be used in this sector.

The study described in the article demonstrates the potential of utilizing optimization algorithms as effective artificial intelligence tools. These algorithms have shown promise in practical applications, particularly in situations where it is necessary to replicate experimental settings quickly, albeit with some degree of approximation, through computer simulations.

As part of future research, we intend to:

- carry out calculations for a more complicated shape and for the case of kappa dependent on temperature,
- take into account a larger number of optimized parameters for swarm algorithms,
- implement parallelized swarm algorithms in conjunction with numerical modeling of casting solidification and cooling,
- experimentally verify the results of calculations for complex shapes.

## Data Availability

The datasets used and/or analyzed during the current study available from the corresponding author on reasonable request.

## References

[1] Meier A, Gonter M, Kruse R (2017). Artificial intelligence for developing an accident severity prediction function. ATZ Worldw..

[2] Hackwood, S. & Beni, G. Self-organization of sensors for swarm intelligence. In *Proceedings 1992 IEEE International Conference on Robotics and Automation* 819–829 vol. 1, 10.1109/ROBOT.1992.220268 (1992).

[3] Karaboga, D. *An idea based on honey bee swarm for numerical optimization* (Tech. Rep, Citeseer, 2005).

[4] Karaboga, D. & Basturk, B. Artificial bee colony (abc) optimization algorithm for solving constrained optimization problems. In *Foundations of Fuzzy Logic and Soft Computing*, 789–798 (Springer, 2007).

[5] Dorigo M, Maniezzo V, Colorni A (1996). Ant system: Optimization by a colony of cooperating agents. IEEE Trans. Syst. Man Cybern. Part B (Cybern.).

[6] Yang X-S (2017). Nature-Inspired Algorithms and Applied Optimization.

[7] Limoncelli TA (2009). The Practice of System and Network Administration.

[8] Karaboga D, Basturk B (2007). A powerful and efficient algorithm for numerical function optimization: Artificial bee colony (abc) algorithm. J. Glob. Optim..

[9] Komar D (2013). A new implementation of the ant algorithm using multiprocessor and distributed processing technology in the navigation system. Sci. Bull. Wroc. Univ. Appl. Inf. Technol. Inf. Technol..

[10] Hetmaniok E (2015). Solution of the two-dimensional inverse problem of the binary alloy solidification by applying the ant colony optimization algorithm. Int. Commun. Heat Mass Transf..

[11] Hetmaniok E, Słota D, Zielonka A (2015). Using the swarm intelligence algorithms in solution of the two-dimensional inverse stefan problem. Comput. Math. Appl..

[12] Górecki J (2021). Preliminary analysis of the sensitivity of the fem model of the process of dry ice extrusion in the die with a circularly converging channel on the changing its geometrical parameters. IOP Conf. Ser. Mater. Sci. Eng..

[13] Berdychowski M, Górecki J, Biszczanik A, Wałȩsa K (2022). Numerical simulation of dry ice compaction process: Comparison of drucker-prager/cap and cam clay models with experimental results. Materials.

[14] Gawrońska E, Dyja R (2018). Numerical calculations of the cast solidification with the complex shape including the movement of the liquid phase. Arch. Found. Eng..

[15] Das R, Mishra SC, Ajith M, Uppaluri R (2008). An inverse analysis of a transient 2-d conduction-radiation problem using the lattice Boltzmann method and the finite volume method coupled with the genetic algorithm. J. Quant. Spectrosc. Radiat. Transf..

[16] Das R, Mishra SC, Uppaluri R (2009). Retrieval of thermal properties in a transient conduction-radiation problem with variable thermal conductivity. Int. J. Heat Mass Transf..

[17] Das R, Mishra SC, Kumar TP, Uppaluri R (2011). An inverse analysis for parameter estimation applied to a non-Fourier conduction-radiation problem. Heat Transf. Eng..

[18] Sczygiol, N. *Numerical Modeling of Thermomechanical Phenomena in the Solidifying Casting and Mold* (Publishing house of the Czȩstochowa University of Technology (in Polish) 2000).

[19] Taler, J. & Duda, P. *Solving Simple and Inverse Heat Conduction Problems* (Scientific and Technical Publishing House (in Polish), 2003).

[20] Dyja R, Gawrońska E, Sczygiol N (2015). The effect of mechanical interactions between the casting and the mold on the conditions of heat dissipation: A numerical model. Arch. Metall. Mater..

[21] Zhou C, Yin K, Cao Y (2018). A novel method for landslide displacement prediction by integrating advanced computational intelligence algorithms. Sci. Rep..

[22] Gerardo, B. & Wang, J. Swarm intelligence. In *Proceedings of the Seventh Annual Meeting of the Robotic Society of Japan* 425–428. 10.1007/978-3-642-58069-7_38 (1989).

[23] Komar D (2013). A new implementation of an ant algorithm using multiprocessor and distributed computing technologies in navigation system. Sci. Bull. Wroc. Sch. Appl. Inform. Inform..

[24] Tomera M (2015). The use of swarm algorithms to optimize parameters in models of control systems. Sci. J. Fac. Electr. Control Eng. Gdansk Univ. Technol..

[25] Hazem, A. & Glasgow, J. Swarm intelligence: Concepts, models and applications. *School Of Computing, Queens University Technical Report*. 10.13140/2.1.1320.2568 (2012).

[26] Geuzaine C, Remacle J-F (2009). Gmsh: A 3-d finite element mesh generator with built-in pre- and post-processing facilities. Int. J. Numer. Methods Eng..

[27] Kodali HK, Ganapathysubramanian B (2012). A computational framework to investigate charge transport in heterogeneous organic photovoltaic devices. Comput. Methods Appl. Mech Eng..

[28] Balay S, Gropp DW, McInnes LC, Smith F, Barry (1997). Efficient Management of Parallelism in Object-Oriented Numerical Software Libraries.

[29] Gawronska, G., Dyja, R., Bȩdkowski, B. & Cyganik, L. Modeling of heat flow using commercial and authorial software on the example of a permanent magnet motor. In *MATEC Web of Conferences* **254**. 10.1051/matecconf/201925402032 (2019).

[30] Dyja R, Gawronska E, Grosser A, Jeruszka P, Sczygiol N (2016). Estimate the impact of different heat capacity approximation methods on the numerical results during computer simulation of solidification. Eng. Lett..

[31] Garnier S, Gautrais J, Theraulaz G (2007). The biological principles of swarm intelligence. Swarm Intell..

[32] Tong A (2001). Improving the accuracy of temperature measurements. Sens. Rev..

[33] Gawrońska E, Dyja R, Zych M, Domek G (2022). Selection of the heat transfer coefficient using swarming algorithms. Acta Mech. et Autom..

[34] Wang Y, Ge J, Miao S, Jiang T, Shen X (2023). Application of hybrid artificial bee colony algorithm based on load balancing in aerospace composite material manufacturing. Expert Syst. Appl..

